# Identification of Differentially Expressed IGFBP5-Related Genes in Breast Cancer Tumor Tissues Using cDNA Microarray Experiments

**DOI:** 10.3390/genes6041201

**Published:** 2015-11-10

**Authors:** Mustafa Akkiprik, İrem Peker, Tolga Özmen, Gökçe Güllü Amuran, Bahadır M. Güllüoğlu, Handan Kaya, Ayşe Özer

**Affiliations:** 1 Department of Medical Biology, School of Medicine, Marmara University, Istanbul 34854, Turkey; E-Mails: pekerirem@gmail.com (İ.P.); gokcegullu@gmail.com (G.G.A.); aozer@marmara.edu.tr (A.Ö.); 2 General Surgery, School of Medicine, Marmara University, Istanbul 34899, Turkey; E-Mails: drtolgaozmen@yahoo.com.tr (T.Ö.); bmgulluoglu@marmara.edu.tr (B.M.G.); 3 Department of Pathology, School of Medicine, Marmara University, Istanbul 34899, Turkey; E-Mail: hkaya@marmara.edu.tr

**Keywords:** gene expression profiling, microarray, pathway analysis, IGFBP5, breast cancer

## Abstract

IGFBP5 is an important regulatory protein in breast cancer progression. We tried to identify differentially expressed genes (DEGs) between breast tumor tissues with IGFBP5 overexpression and their adjacent normal tissues. In this study, thirty-eight breast cancer and adjacent normal breast tissue samples were used to determine IGFBP5 expression by qPCR. cDNA microarrays were applied to the highest IGFBP5 overexpressed tumor samples compared to their adjacent normal breast tissue. Microarray analysis revealed that a total of 186 genes were differentially expressed in breast cancer compared with normal breast tissues. Of the 186 genes, 169 genes were downregulated and 17 genes were upregulated in the tumor samples. KEGG pathway analyses showed that protein digestion and absorption, focal adhesion, salivary secretion, drug metabolism-cytochrome P450, and phenylalanine metabolism pathways are involved. Among these DEGs, the prominent top two genes (MMP11 and COL1A1) which potentially correlated with IGFBP5 were selected for validation using real time RT-qPCR. Only COL1A1 expression showed a consistent upregulation with IGFBP5 expression and COL1A1 and MMP11 were significantly positively correlated. We concluded that the discovery of coordinately expressed genes related with IGFBP5 might contribute to understanding of the molecular mechanism of the function of IGFBP5 in breast cancer. Further functional studies on DEGs and association with IGFBP5 may identify novel biomarkers for clinical applications in breast cancer.

## 1. Introduction

The insulin-like growth factor (IGF) signaling pathway has an important role in cell growth, differentiation, apoptosis regulation and tumor development [1]. The IGF axis comprises two growth factors (IGF-I, IGF-II), two IGF receptors (IGF-IR, IGF-IIR), and seven IGF-binding proteins (IGFBPs) which regulate the mitogenic activities of the IGFs, a group of IGFBP-related proteins that bind IGFs with low affinity and IGFBP proteases. IGFBP5 is the most conserved binding protein of the IGFBP family in all vertebrates and is frequently dysregulated in human cancers.

IGFBP5 has numerous functional roles in carcinogenesis and it acts both IGF-dependent and independent mechanisms [2]. There are many findings and assumptions about the role of IGFBP5 and it is very crucial to identify the role of IGFBP5 in cancer progression, especially in breast cancer. Some studies concluded that IGFBP5 acts as an oncogene, since the high protein level is linked to proliferation, metastasis, poor prognosis, drug sensitivity and limited response to endocrine treatment, but on the other hand some studies revealed that IGFBP5 acts as a tumor suppressor gene due to being related with apoptotic, anti-metastatic, and anti-migratory effects and good outcomes [1].

Some studies indicated that IGFBP5 expression is high in tumors and considered IGFBP-5 as having pro-metastatic capacity [3,4]. Exogenous IGFBP5 has been shown to have a protective effect for ceramid-induced apoptosis [5]. IGFBP5 accumulates in the cytoplasm and is related with bad prognoses in the breast cancer tissue [6]. Our group previously reported that apoptotic and migratory potential of IGFBP5 depends on cellular localization which is regulated by nuclear localization signal into C-terminal domain of the protein in breast cancer cells [7]. Overexpression of IGFBP5 has been found to be associated with poor outcomes of breast cancer patients [8]. A recently published study indicated the important role of IGFBP5 in tumor progression in urothelial carcinoma and associated IGFBP5 over-expression with advanced tumor stage, frequent mitosis and poorer clinical outcomes [9].

A recently published genome-wide association study (GWAS) shows that the 2p35 locus is an important risk factor for breast cancer [10]. Furthermore, an SNP in the locus rs4442975 (G/T) has been reported to contribute to changes in IGFBP5 expression. The data suggested that the G-allele of rs4442975 refers to increased breast cancer susceptibility through reduced IGFBP5 expression. IGFBP-5 expression was also shown to be correlated with increased survival rate and to help maintaining tumor sensitivity to tamoxifen in breast cancer patients [11]. The same study showed over-expression of IGFBP-5 in MCF-7 xenografts inhibited tumor development in mice. In other recent studies the tumor suppressant role of IGFBP-5 has been shown in melanoma cells [12], osteosarcoma [13] and ovarian cancer cells [14]. IGFBP5 has been also reported to suppress cell growth and cause G2/M arrest in PANC-1 pancreatic cancer cells [15]. Sureshbabu *et al.* [16] verified that IGFBP-5 increases epithelial cell adhesion to the extracellular matrix (ECM) in MCF-7 human breast cancer cells and at the same time inhibits migration by maintaining E-cadherin expression. Sureshbabu *et al.* assume that this is how IGFBP5 plays a key role in preventing metastasis. One of the latest reports on the subject showed that over-expression of IGFBP5 levels inhibited the epithelial-mesenchymal transition (EMT) and decreased E-cadherin expression and the key stem cell markers NANOG, SOX2, OCT4, KLF4, and CD133 in human melanoma cell line [12].

Besides the anti-apoptotic effects, IGFBP5 was shown to have stimulatory effects on apoptosis in different cancer types. Overexpression of IGFBP5 is known to inhibit IGF-I activation of IRS-1 (IR substrate-1), FKHRL-1 (forkhead in rhabdomyosarcoma-like 1) and protein kinase B. This indicates that IGFBP5 acts as an apoptotic factor by inhibiting the action of local IGF-I [17]. Butt [18] *et al.* found that IGFBP5 activates caspase 8 and 9 MDA-MB-231 breast cancer cell line, which leads to apoptosis through Bcl-2 in the intrinsic apoptotic pathway. The apoptosis-inducing role of IGFBP5 has also been shown in prostate cells [19] and ovarian cells [20]. Recently it was reported that IGFBP5 intercedes neuronal apoptosis through the regulation of mitochondrial cytochrome c release and caspase 3 activation [21].

The functional and clinical meaning of expressional differences of IGFBP5 in tumor tissue is still controversial. To reveal differences in the gene expression profile between IGFBP5 overexpression in breast cancer tissues compared to matched normal breast tissue, a cDNA microarray experiment was conducted. Importantly, one of the top differentially expressed genes (DEGs), COL1A1, was validated by real time RT-qPCR and a positive correlation with IGFBP5 overexpression was found. In addition, there was one more positive correlation between the expression of COL1A1 and MMP11. Therefore, our results suggest that these two genes work together coordinately and contribute to breast cancer progression.

## 2. Experimental Section

### 2.1. Clinicopathological Parameters of Breast Cancer Patients

In total, 38 breast cancer patients, who were diagnosed at the Department of General Surgery, Marmara University School of Medicine from July 2010 to January 2012, were included in this study. Patients were between the ages of 38 and 73 and the median age was 59. Patients’ ages, histologic types, tumor grades, molecular subtypes, hormone statuses (Her2, ER, PR), proliferation marker Ki67 statuses, menopausal statuses, numbers of pregnancies, ages of menarche, tumor sizes and localizations have been recorded (Table 1). Human epidermal growth factor receptor 2 (Her2), progesterone (PR), estrogen (ER) receptor and Ki67 statuses were evaluated by means of immunohistochemical staining with specific monoclonal antibody. Moreover, if the Her2 score was 2, fluorescence *in situ* hybridization (FISH) was performed to clarify the Her2 status. Molecular subtypes were determined by using Goldhirsch classification [22]. The study protocol was approved by the Local Ethics Committee of Marmara University (Istanbul, Turkey).

**Table 1:** Differences of clinicopathological parameters depending on tumoral expression of IGFBP5 compared to adjacent normal tissue.

Characteristics	Tumoral Expression of IGFBP5 Compared to Adjacent Normal Tissue		*p*-Value
	High (n = 21)	Low (n = 17)	
Age (years)	55.66	55.52	0.9684
Menarche age (years)	12.95	14.18	0.0149
Pregnancy (number)	3.66	3.11	0.5876
Menopausal state			
Pre	7	3	ns
Peri	2	1	
Post	12	13	
Tumor size (mm)	26.71	23	0.3578
Tumor localization			
Left	10	13	ns
Right	10	4	
Bilateral	1	0	
Estrogen receptor status			
Positive	16	13	ns
Negative	5	4	
Progesterone receptor status			
Positive	14	13	0.7210
Negative	7	4	
Her2 status			
Positive	5	2	0.4267
Negative	16	15	
Ki67 status			
Positive	11	12	0.3264
Negative	10	5	
*Tumor grade*			
1	0	5	0.0123
2&3	21	12	
*Tumor histology*			
IDC	15	11	ns
ILC	1	0	
IMC	3	3	
Others	2	3	
*Molecular subtypes*			
Luminal A	6	5	ns
Luminal B Her2(−)	4	7	
Luminal B Her2(+)	6	1	
Erb-B2 overexpression	1	2	
Basal-like	4	2	

### 2.2. RNA Isolation and Quantification of IGFBP5 Expression in Breast Cancer Using qPCR

RNA was isolated from fresh frozen breast cancer and adjacent normal breast tissues from the 38 cases were isolated with High Pure RNA Tissue Kit according to instruction protocol (Roche, Mannheim, Germany). Transcriptor High Fidelity cDNA synthesis kit (Roche) was used for cDNA synthesis with 500 ng of total RNA in a reaction volume of 20 µL. Real time qRT-PCR was performed in 20 µL of final reaction volume with 5 µL of cDNA, 10 µL LightCycler 480 Probes Master mix, and 2 µL of primer-probe mix and 3 µL water to complete final volume. Reactions were carried out under the following cycling conditions: 5 min at 95 °C for pre-incubation, 10 s at 95 °C, 30 s 60 °C, 1 s 72 °C for amplification with 45 cycles, 10 s at 40 °C for cooling. All reactions were performed in duplicate for reference housekeeping gene, beta actin and IGFBP5 by using LightCycler 480 instrument. Relative quantification was calculated by delta-delta Ct method, subsequent to IGFBP5 expression normalization to beta actin expression.

### 2.3. Selection of Samples and Coding for Microarray

Of 38 samples for which we analyzed IGFBP5 expression level both in tumor and adjacent normal tissues, we selected those with the first 6 highest IGFBP5 expression in tumor samples compared to normal. T codes represent tumor tissues, N codes represent normal tissues. Microarray data analysis was excluded from one sample because it was found to be under the criteria for analysis. Finally, five tumor samples formed a group and five normal tissues formed another group. Examples belonging to the same group were evaluated as biological replicates. Tumor characteristics and fold changes of IGFBP5 expression in patients for use in microarray analysis are presented in Table 2.

**Table 2:** Tumor characteristics and fold changes of IGFBP5 expression in patients for use in microarray analysis.

Patients Code	Age	ER	PR	Her2	Histology	Molecular Subtypes	Stage	Size (mm)	Fold Changes of IGFBP5
T1	47	60%	60%	(neg.)	ILC	lumA	T1N1	15	2.276
T2	73	50%	10%	(neg.)	IDC	lumA	T2N3	23	16.528
T3	40	90%	90%	(neg.)	IMC	lumB	T2N2	30	3.233
T4	42	90%	60%	(neg.)	IDC	lumA	T2N0	22	2.022
T5	67	70%	70%	(neg.)	IDC	lumA	T2N2	30	4.892

### 2.4. Gene Expression Profiling by Microarray and Validation with qPCR

RNA quality of samples was carried out with Agilent Bioanalyzer. Gene expression profiling of highly expressed IGFBP5 in tumor tissue compared to their matching adjacent normal tissue were performed by using a humanHT-12 v4 expression bead chip (Illumina, CA, USA). Each bead chip has more than 47,000 probes derived from the National Center for Biotechnology Information Reference Sequence (NCBI) RefSeq Release 38 (7 November 2009) and other sources. The microarray data was converted into electronic data by means of the GenomeStudio program. Signal intensity graphics of microarray data before normalization were generated for quality control purposes. The background noise was removed from each sample for comparative data analysis and data percentages of all samples (quantile) were subjected to normalization. Then, for use in the data analysis step, log transformation was conducted on the data that has been normalized. Hierarchical clustering analysis of the whole genome gene expression profiles of the samples was carried out employing a “Euclidean-average” approach.

Validation of microarray results was accomplished by performing qRT-PCR on COL1A1 and MMP11 genes in 37 patients. The expression levels of the target genes in each sample were calculated with a ∆∆Ct method. Beta actin was used as a housekeeping gene and the relative quantification was done by normalizing the cancer tissue data to its adjacent normal tissue data. All qRT-PCR experiments were performed in duplicate.

### 2.5. Pathways Analysis

Pathways analysis was performed using a hypergeometric test method; the *p*-value was been calculated and modified according to the Benjamin-Hochberg procedure. The adjusted *p*-value = 0.05 filter states the statistical significance that is used to determine the linked pathway, the condition that at least two genes must show different expressions.

### 2.6. Statistical Analysis

Student’s t tests (unpaired, two-tailed), Fisher’s exact probability test or the chi-square test were used to evaluate possible associations between the mRNA expression of IGFBP5 and various clinicopathological parameters. The data obtained after completion of the preliminary analysis used for the differential expression analysis was conducted to the data obtained after completion of the preliminary analysis. A SAM (Significance Analysis of Microarrays) method was used for determining the probe indicating expression of different genes. The analysis was conducted by comparing the probes’ expression levels in the normal tissue group and the tumor tissue group. FC-fold change and *p*-value for each probe were calculated statistically. After the statistical comparisons between the two groups, values of the probes that had FDR (False Discovery Rate) ≤ 0. 05 and FC (Fold Change) ≥ 1.5 were identified. Correlation between the mRNA expression levels were calculated using Spearman’s correlation. *p-*values ˂ 0.05 were considered statistically significant.

## 3. Results

### 3.1. IGFBP5 mRNA Expression Levels in Breast Cancer Patients

IGFBP5 levels were detected to be upregulated in 21 (55.3%) and downregulated in 17 (44.7%) tumor samples compared to their adjacent normal tissues. The mean and median levels of IGFBP5 expression were in high and low expressed groups 4.2 and 0.53 fold for mean and 2.9 and 0.55 fold for median, respectively. IGFBP5 expression level was not statistically different between tumor and normal tissue (*p* = 0.2125). Overexpression of IGFBP5 was more common in patients with advanced tumor grades (*p* = 0.0123). Menarche age was found to be lower in patients with IGFBP5 overexpression (*p* = 0.0149).

### 3.2. Differentially Expressed Genes and Hierarchical Clustering of Microarray Data

**Figure 1:**
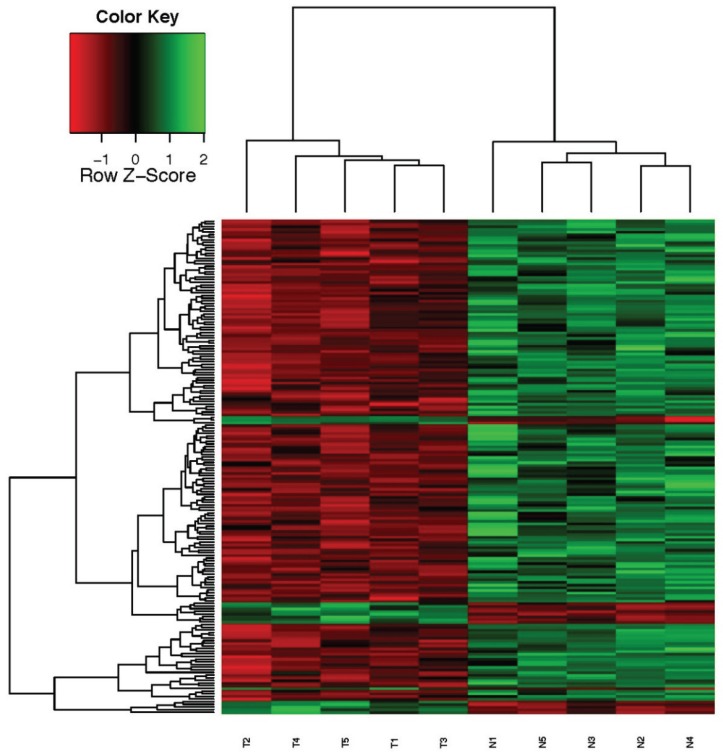
Heat map of cDNA microarray expression data from breast cancer patients with overexpressed IGFBP5 in tumor tissue compared to adjacent normal tissue.

Microarray analysis revealed that there were 186 probes that showed significantly different expression in breast cancer tissues compared to normal breast tissues. Of the 186 genes, 17 genes were upregulated (Table 3) and 169 genes were downregulated (Table 4) in tumor samples. Probes that were found with significantly different expression between two groups were used for hierarchical clustering and the samples were grouped according to their expression patterns. Clustering results and the expression scales are shown in the heat map (Figure 1). Our microarray data also proved that IGFBP5 was one of the upregulated genes in all the breast cancer samples compared with their adjacent normal tissues (*p* = 0.0004, median difference = 1.379).

The expression of genes is hierarchically clustered on the y axis, and breast cancer tissues and their adjacent healthy normal breast tissue samples are hierarchically clustered on the x axis. The relative gene expression is depicted according to the color scale shown in the left corner. Numbers with T indicate cancer samples; numbers with N indicate healthy control samples.

**Table 3:** List of genes upregulated in the breast cancer group compared with normal breast tissues.

Gene Symbol	RefSeq	Fold Change	Q-Value
CST1	NM_001898.2	58.11773612	5.88 × 10^−6^
MMP11	NM_005940.3	41.38923113	1.08 × 10^−5^
COL1A1	NM_000088.3	20.28765826	3.05 × 10^−5^
GRIA2	NM_000826.2	19.475709	4.92 × 10^−5^
COL5A2	NM_000393.3	8.154198414	4.28 × 10^−5^
SPOCK1	NM_004598.3	7.717882382	5.69 × 10^−5^
NKAIN1	NM_024522.1	5.686495097	3.71 × 10^−5^
DSCR6	NM_018962.1	4.853586146	4.65 × 10^−5^
CLGN	NM_004362.1	4.372809335	7.25 × 10^−5^
KCNF1	NM_002236.4	4.03710734	4.96 × 10^−6^
SLC44A4	NM_025257.2	3.372736146	3.20 × 10^−5^
SLC44A4	NM_032794.1	3.353011207	2.99 × 10^−5^
FAM83D	NM_030919.2	3.063515303	4.59 × 10^−5^
ESM1	NM_007036.3	2.393905549	3.23 × 10^−5^
NINJ1	NM_004148.3	2.24489298	6.78 × 10^−5^
LCLAT1	NM_001002257.1	1.733148032	6.39 × 10^−5^
SLC12A8	NM_024628.4	1.699383326	6.41 × 10^−5^

**Table 4:** List of genes downregulated in the breast cancer group compared with normal breast tissues.

Gene Symbol	RefSeq	Fold Change	Q-Value
DST	NM_001723.4	−10.5318799	3.31 × 10^−6^
OXTR	NM_000916.3	−11.0105517	2.19 × 10^−5^
COL17A1	NM_000494.3	−11.04207984	3.03 × 10^−5^
HAS3	NM_005329.2	−11.24842575	7.35 × 10^−7^
SAA1	NM_199161.1	−11.59121389	1.25 × 10^−5^
KRT17	NM_000422.1	−12.70489015	3.84 × 10^−5^
ACTG2	NM_001615.3	−14.48863197	1.91 × 10^−5^
KRT14	NM_000526.3	−15.692027	2.20 × 10^−6^
KRT5	NM_000424.3	−15.77641561	2.44 × 10^−5^
KRT15	NM_002275.2	−16.38531094	1.6 × 10^−4^
SYNM	NM_015286.5	−17.22861604	5.91 × 10^−5^
PPP1R1B	NM_181505.1	−17.74088495	1.65 × 10^−6^
KLK7	NM_005046.2	−17.88824515	6.61 × 10^−6^
SOX10	NM_006941.3	−19.24267129	1.84 × 10^−7^
KLK5	NM_001077491.1	−20.72022752	3.53 × 10^−5^
KRT6B	NM_005555.3	−21.63963922	1.82 × 10^−5^
STAC2	NM_198993.2	−21.88100857	9.37 × 10^−6^
KLK5	NM_012427.4	−22.06231675	4.41 × 10^−6^
MUCL1	NM_058173.2	46.66120963	2.7 × 10^−4^

Only the results were listed as average ratio > 10.0

### 3.3. Analysis of KEGG Pathways

There are 186 probes that show different expression levels between two groups that are statistically significant. These 186 probes were assigned to their associated genes and the databases was searched for the linked pathways. As a result, the expression level of 149 unique genes code was assigned to at least one common pathway. The identified 149 genes were used to test any pathway significantly enriched in the status number and gene expression changes, having taken into consideration the 29.345 reference gene on Illumina Human HT-12V4A microarray system. According to the operating parameters, five pathways and related genes were found to significantly vary between the two groups (Table 5 and Table 6).

**Table 5:** KEGG pathway analysis of DEGs.

Pathway	Reference Genes in Category	Expected Number in the Category	Gene Count	Enrichment Ratio	Raw p-val	Adjusted *p*-val
Protein digestion and absorption	77	0.58	5	8.62	0.0003	0.0087
Focal adhesion	184	1.39	6	4.33	0.0027	0.0217
Salivary secretion	79	0.6	4	6.72	0.003	0.0217
Drug metabolism	72	0.54	4	7.37	0.0021	0.0217
Phenylalanine metabolism	17	0.13	2	15.62	0.0071	0.0412

**Table 6:** KEGG pathway analysis of DEGs and their fold changes in microarray data.

Pathway	Gene Symbol	Gene Name	Fold Change
Protein digestion and absorption	COL17A1	Collagen, type XVII, alpha 1	−8.19
	COL5A2	Collagen, type V, alpha 2	8.15
	COL1A1	Collagen, type I, alpha 1	20.29
	KCNN4	Potassium intermediate/small conductance calcium-activated channel, subfamily N, member 4	−5.43
	MME	Membrane metallo-endopeptidase	−7.67
Focal adhesion	COL5A2	Collagen, type V, alpha 2	8.15
	MET	Met proto-oncogen (hepatocyte growth factor)	−2.43
	LAMC2	Laminin, gamma 2	−2.61
	COL1A1	Collagen, type I, alpha 1	20.29
	CAV2	Caveolin 2	−3.37
	MYLK	Myosin light chain kinase	−6.93
Salivary secretion	TRPV6	Transient receptor potential cation channel, subfamily	−3.64
	CST1	Cystatin SN	58.12
	KCNN4	Potassium intermediate/small conductance calcium-activated channel, subfamily N, member 4	−5.43
	RYR3	Ryanodine receptor 3	−4.76
Drug metabolism cytochrome P450	FMO2	Flavin containing monooxygenase 2 (non-functional)	−5.23
	GSTP1	Glutation S-transferase pi 1	−3.79
	ALDH3A1	Aldehyde dehydrogenase 3 family, member A1	−2.86
	ALDH1A3	Aldehyde dehydrogenase 1 family, member A3	−9.46
Phenylalanine metabolism	ALDH3A1	Aldehyde dehydrogenase 3 family, member A1	−2.86
	ALDH1A3	Aldehyde dehydrogenase 1 family, member A3	−9.46

### 3.4. Advanced Level Bioinformatics

**Figure 2:**
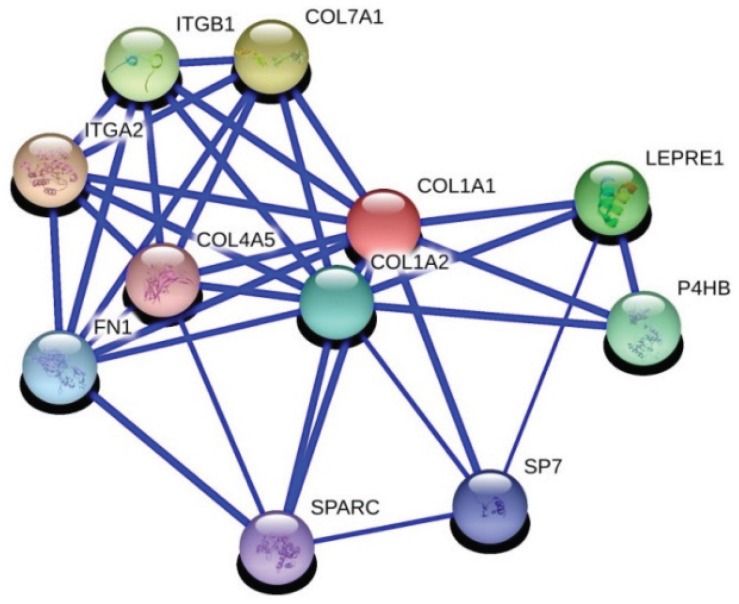
Evidence views of COL1A1 gene and its association with FN1 by using STRING 10.

We used the ExPASy bioinformatics resource portal and MiMI (Michigan molecular interactions) database for differentially expressed genes and their interactions and pathways considered with IGFBP5. Three genes, CST1 (gene id: 1469), MMP11 (gene id: 4320) and COL1A1 (gene id: 1277), which are involved in the highest increased genes within the differentially expressed genes list, came into prominence. CST1 plays a critical role in cancer progression and metastasis. MMP11 is an important cancer metastasis regulatory protein. On the other hand, COL1A1 is a main extracellular matrix protein and it also interacts with one of the IGFBP5 related proteins, FN1 (fibronectin 1, gene id: 2335). The COL1A1 gene and its interactions are shown in Figure 2. We can speculate that these three genes could be possibly playing a critical role in the molecular mechanism of the metastatic potential of IGFBP5 in breast cancer.

### 3.5. Validation Experiments of COL1A1 and MMP11 with IGFBP5 by Real Time qPCR

**Figure 3:**
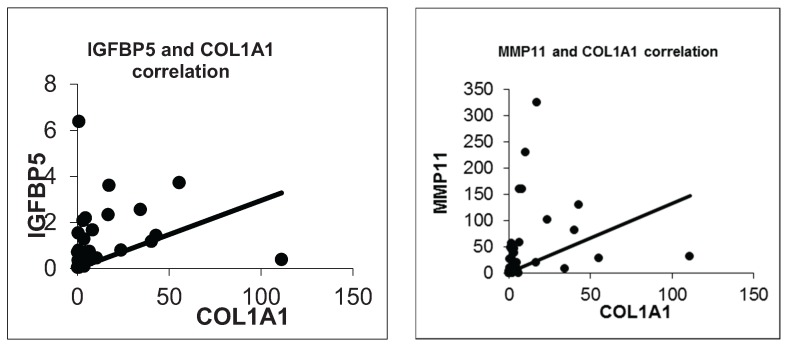
Scatter plots of the selected genes in real time qPCR.

According to Spearman’s rho correlation test, IGFBP5 and COL1A1, COL1A1 and MMP11 were significantly positively correlated (R = 0.476, *p* = 0.003 and R = 0.602, *p* = 0.000, respectively). The scatter diagram of the selected genes is shown in Figure 3. Unlike the microarray data, MMP11 and IGFBP5 did not show significant positive correlation (R = 0.226, *p* = 0.199).

## 4. Discussion

IGFBP5 is one of the family members of IGF-binding proteins which play a critical role in tumor progression, especially cell survival, death and metastasis processes. The functional role of fluctuational IGFBP5 expression in origination and development of different types of cancer is still mysterious. IGFBP5 has diverse effects on growth of cancer cells depending on cell type and cell content. Because of the different functions of IGFBP5, a microarray system method, which provides a global picture in the cells, is the best method to clarify and discover new correlations with the genes. Using this method, we firstly determined potentially IGFBP5-related genes by using the microarray system in samples of breast cancer patients.

In this study, we identified that top differentially increased gene expressions with IGFBP5 are CST1, MMP11 and COL1A1. We focused on these three genes because of their associations and potential roles in extracellular matrix (ECM)-mediated pathways and metastasis process in cancer. Our previously published paper has shown the role of IGFBP5 on metastatic capacity of breast cancer [7]. In the present study, KEGG pathway analysis reveals that focal adhesion and drug metabolism pathways are correlated with IGFBP5 expressional differentiation. These two pathways play a pivotal role in understanding cancer progression and development of treatment strategies considering IGFBP5.

Our microarray data shows that CST1 (cystatin SN) is one of the top DEGs in IGFBP5 overexpressed group. The general function of cystatins is the inhibition of the proteolytic activity of cysteine proteases which are involved in migration of cancer cells. Recent findings show that CST1 was highly expressed in colon, gastric and pancreatic cancers. CST1-overexpressing colon cancer cell lines exhibited increased tumor growth as well as metastasis in a xenograft nude mouse model [23]. Overexpression of CST1 was also correlated with malignancy-associated proteins such as PCNA, cyclin D1, cyclin A2 and cyclin E in pancreatic cancer cell line [24]. And finally, suppression of metastasis has been found to be correlated with reduced expression of CST1 in breast cancer cell line [25]. We can suggest that metastatic potential of IGFBP5 might be correlated with CST1.

Extracellular matrix genes are critical players in tumor progression and metastasis. Here we identified an important correlation between IGFBP5 and two regulators of ECM-related pathways using gene co-expression analysis. The first is matrix metalloproteinase-11 (MMP11), which is related with distance metastasis [26], resistance to anoikis [27] and poor outcomes [28] in breast cancer. The second is collagen 1a1 (COL1A1), which is an important regulator of pro-metastatic processes in cancer. Interactions with fibronectin and collagen 1a1 has been identified in breast cancer and related with adhesion of breast cancer in a previous study [29]. We also know that IGFBP5 interacts with fibronectin and regulates cell migration [30].

We also confirmed a positive correlation between IGFBP5 and COL1A1 expression (R = 0.476, *p* = 0.003). Besides that COL1A1 and MMP11 were significantly positively correlated (R = 0.602, *p* = 0.000). So we anticipate that IGFBP5 could be a possible modifier on metastatic capacity of breast cancer through regulating COL1A1 or MMP11. Further functional and protein-protein interactions studies are needed to clarify a likely correlation between IGFBP5, COL1A1 and MMP11 considering the metastasis process in breast cancer.

A recently published whole-genome analysis study identified a new breast cancer risk locus, 2q35. Fine scale mapping of the locus shows the strongest candidate for causality, SNP rs4442975, flanks a transcriptional enhancer that physically interacts with the promoter of IGFBP5. Furthermore, presence of this polymorphism reduced IGFBP5 expression [10]. Whereas, we did not reach statistically significant difference between tumor and normal tissue samples from breast cancer patients considering IGFBP5 expression (*p* = 0.2125), we found a positive correlation between higher expression of IGFBP5 and advanced stage of breast cancer. Further studies with a large sample size are needed to clarify tumoral expression of IGFBP5 and its clinical significance in breast cancer.

## 5. Conclusions

In conclusion, functional role of tumoral IGFBP5 expression is still controversial. We did not find a statistically significant increase in IGFBP5 in breast cancer tissues compared to adjacent normal tissue, but higher expression of IGFBP5 was correlated with advanced tumor grades (*p* = 0.0123). To understand differences of gene expression profile in samples with high tumoral expressions of IGFBP5 compared to matching normal breast tissue, cDNA microarray experiment was conducted. We determined that the top differentially expressed genes with coordinal expressed IGFBP5 were CST1, MMP11 and COL1A1. These three genes play a pivotal role in cancer progression and metastasis processes. Validation of a limited number of the genes shows that some of these genes could be potentially affecting IGFBP5 function about the processes. We can suggest that IGFBP5 may directly or indirectly regulate cancer metastasis pathways through the top differentially expressed genes. Further studies with a large sample size and functional experiments are needed to clarify importance of tumoral IGFBP5 expression and its molecular function in breast cancer metastasis.
